# An efficient quasi-Monte Carlo method with forced fixed detection for photon scatter simulation in CT

**DOI:** 10.1371/journal.pone.0290266

**Published:** 2023-08-24

**Authors:** Guiyuan Lin, Shiwo Deng, Xiaoqun Wang

**Affiliations:** 1 School of Mathematics and Statistics, Hunan First Normal University, Changsha, China; 2 National Center for Applied Mathematics, Southern University of Science and Technology, Shenzhen, China; 3 Department of Mathematical Sciences, Tsinghua University, Beijing, China; Tel Aviv University, ISRAEL

## Abstract

Detected scattered photons can cause cupping and streak artifacts, significantly degrading the quality of CT images. For fast and accurate estimation of scatter intensities resulting from photon interactions with a phantom, we first transform the path probability of photons interacting with the phantom into a high-dimensional integral. Secondly, we develope a new efficient algorithm called gQMCFFD, which combines graphics processing unit(GPU)-based quasi-Monte Carlo (QMC) with forced fixed detection to approximate this integral. QMC uses low discrepancy sequences for simulation and is deterministic versions of Monte Carlo. Numerical experiments show that the results are in excellent agreement and the efficiency improvement factors are 4 ∼ 46 times in all simulations by gQMCFFD with comparison to GPU-based Monte Carlo methods. And by combining gQMCFFD with sparse matrix method, the simulation time is reduced to 2 seconds in a single projection angle and the relative difference is 3.53%.

## Introduction

Computed tomography (CT) has been widely used in many fields, and has revolutionized diagnostic radiology with its great advantages of non-destructive, non-overlapping images and high-resolution since the first CT was developed in 1972 by Hounsfield [1, 2].

**Fig 1:**
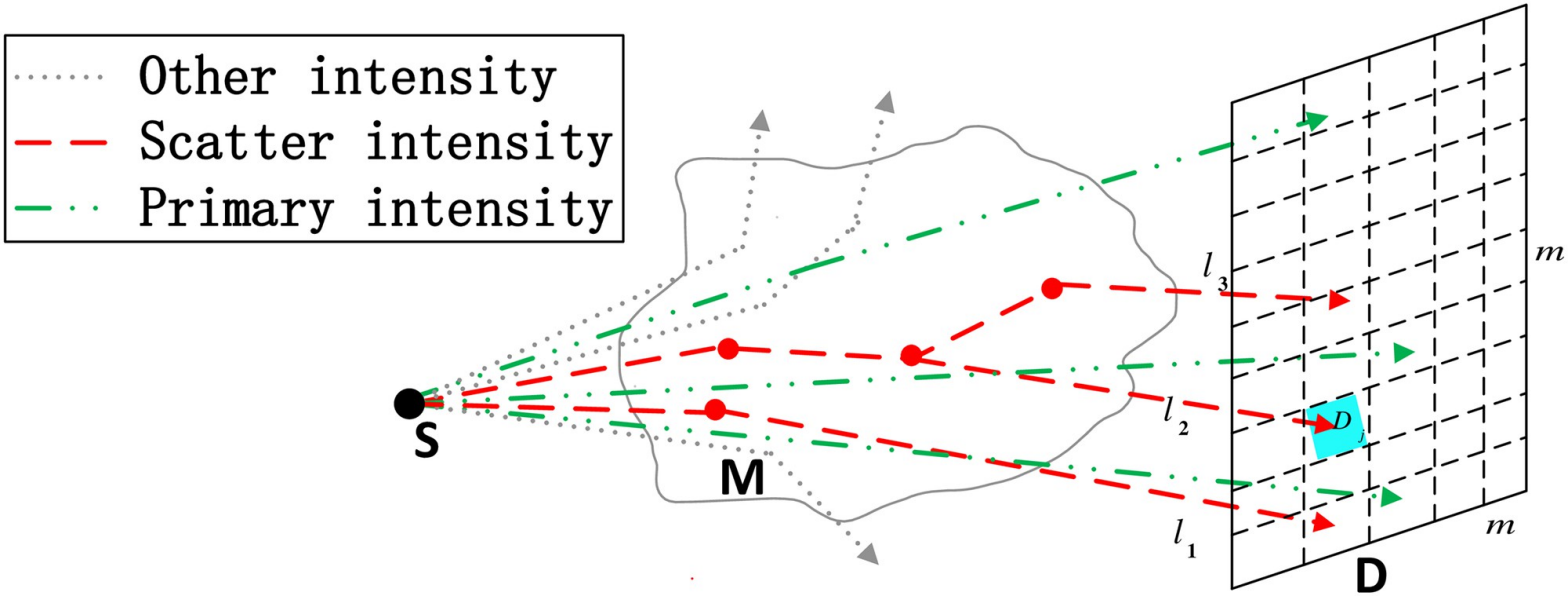
An illustration of photon paths in CT.

The process of CT image reconstruction produces an estimate of the linear attenuation coefficient *μ*_*tot*_(***x***, *E*) of the phantom M from data obtained by measuring the attenuation of X-rays along multiple paths through M, where ***x*** is the spatial coordinates and *E* is the photon energy. Let *I*_0_(*l*) and *I*(*l*) represent the intensity of the X-ray entering and exiting M along the path *l*, respectively. Both scatter and primary intensities are recorded by the detector D. An illustration of photon paths in CT is shown in Fig 1. The CT system is composed of X-ray source S, a three-dimensional phantom M and a detector D, which is composed of *m* × *m* detector pixels. Photons emitted from the X-ray source S will be absorbed or scattered when passing through the phantom M. After a certain amount of scatter, photons will reach the detector D or not with a certain probability. As shown in Fig 1, the red lines *l*_1_, *l*_2_, *l*_3_ are 1-, 2-, 3-order scatter photon paths, respectively, and they reach the detector D. The green lines are the primary intensities which are useful. The gray lines are the other intensities that don’t reach the detector D.

The Lambert-Beer law [3] describes the attenuation of X-rays passing through the measured phantom M under ideal conditions, without considering scattered photons that may reach the detector. By the Lambert-Beer law

$$\begin{array}{c} I\left(l\right)=I_{0}\left(l\right)\text{exp}[-\int_{l}\mu_{tot}\left(x,E\right)\text{d}x], \end{array}$$ (1)

so *μ*_*tot*_(***x***, *E*) of each projection data will be underestimated when scatter is present. Scatter intensities induce nonlinear errors in the measurement of *μ*_*tot*_ values and have a complex effect on the reconstructed CT images. It may lead to dark steak artifacts between image regions of high attenuation, cup-shaped artifacts in homogeneous objects and the reduction of the contrast resolution of the reconstructed CT images [4]. Furthermore, the contribution of scatter intensity to the total intensity (which is the sum of the scatter and primary intensity) concurrently grows when using high-energy X-ray, or multi-row and flat-panel detectors in CT [5]. Therefore, superior scatter correction algorithms are needed.

Numerous methods for scatter calculation and correction have been proposed, such as scatter kernel superposition (SKS) algorithms [6], fast adaptive SKS (fASKS) [7], Monte Carlo (MC) methods [5, 8–13], Acuros CTS (deterministic solution of linear Boltzmann transport equation) [14, 15], neural network approaches [16, 17] and so on. SKS uses scatter point spread functions generated from pencil beams to perform deconvolution on the measured projection data using derived kernels. Although SKS is an approximate method for scatter estimation with advantages such as high computational efficiency and practicality, it suffers from inaccurate estimation of scatter intensities. MC method has been widely applied in radiation therapy, medical imaging and nuclear medicine for solving a variety of radiation transport problems [18]. Many MC-based scatter correction tools have been developed, such as PENELOPE [8], methods of combining MC and fixed forced detection (FFD) [5, 9, 10], which only consider theoretical interacting photons to make a contribution to the intensity in the fixed detector pixel, and GPU-based MC tools (denoted by MC-GPU [11], gMCDRR [12] and gMMC [13]). MC-GPU, which is a GPU implementation of PENELOPE, is expected to have the same accuracy as standard MC. Compared with MC-GPU, gMCDRR uses the differential cross section (DCS) data to calculate the scatter direction and uses the generalized inverse transform method instead of the acceptance-rejection method to sample the scatter direction. gMMC is a GPU-based Metropolis MC, which uses the Metropolis-Hasting algorithm to sample the whole photon paths from the X-ray source to the detector. However, GPU-based MC tools has a low convergence rate *O*(*N*^−1/2^), where *N* is the number of simulations. When greater accuracy is required, *N* needed increases rapidly. This causes high computational burden. Addtionally, the existing GPU-based MC tools passively receives the scatter intensity reaching the detector, leading to significant computational effort being spent on simulating those photons that do not contribute to the scatter intensity, therefore reducing the overall computational efficiency. Although gMMC can improve photon utilization, it brings serious computational burden and other new problems. Acuros CTS estimated scatter intensities in X-ray projection data by deterministically solving the linear Boltzmann transport equation (LBTE) and was applied to the clinic for treatment planning for radiotherapy of cancer. Although different scatter estimation and correction approaches have been developed, a standard solution is still being studied.

Quasi-Monte Carlo (QMC) method is a deterministic version of MC method which uses low discrepancy sequences [19, 20] instead of random sequences for simulation. Under suitable conditions, the convergence rate of QMC is close to *O*(*N*^−1+*ϵ*^), *ϵ* > 0, which is asymptotically faster than that of MC for a fixed dimension. QMC is usually more efficient than MC for high-dimensional integration. gQMCFRD, which combines QMC with forced random detection (FRD), was proposed recently [21]. The efficiency improvement factors (EIFs) are 27 ∼ 37 times when gQMCFRD is compared to MC-GPU.

The concerns of the paper are to develop and study an efficient scatter estimation and correction mechanism for CT which is suitable for QMC. The contributions of the paper are twofold. Firstly, we transform the problem of simulating the photon scatter in CT into a high-dimensional integral. Secondly, a new and efficient scatter estimation algorithm is developed, which combines GPU-based QMC with forced fixed detection (FFD). Numerical results show that a substantial gain in efficiency can be realized for simulating the scatter intensity in CT relative to MC-based algorithms.

The rest of this paper is organized as follows. The formulation of simulating the photon scatter in CT and the algorithm are introduced in Materials and Methods. The results of the scatter intensities in both a homogeneous Al phantom, Bone-Tissue (BT) cylinder, Shepp-Logan (SL) phantom and Head phantom are presented in Numerical Experiments. Finally, the paper is summarized in Conclusions.

## Materials and methods

### Derivation of photon path probability *P*(*l*_*i*,*j*_)

**Fig 2:**
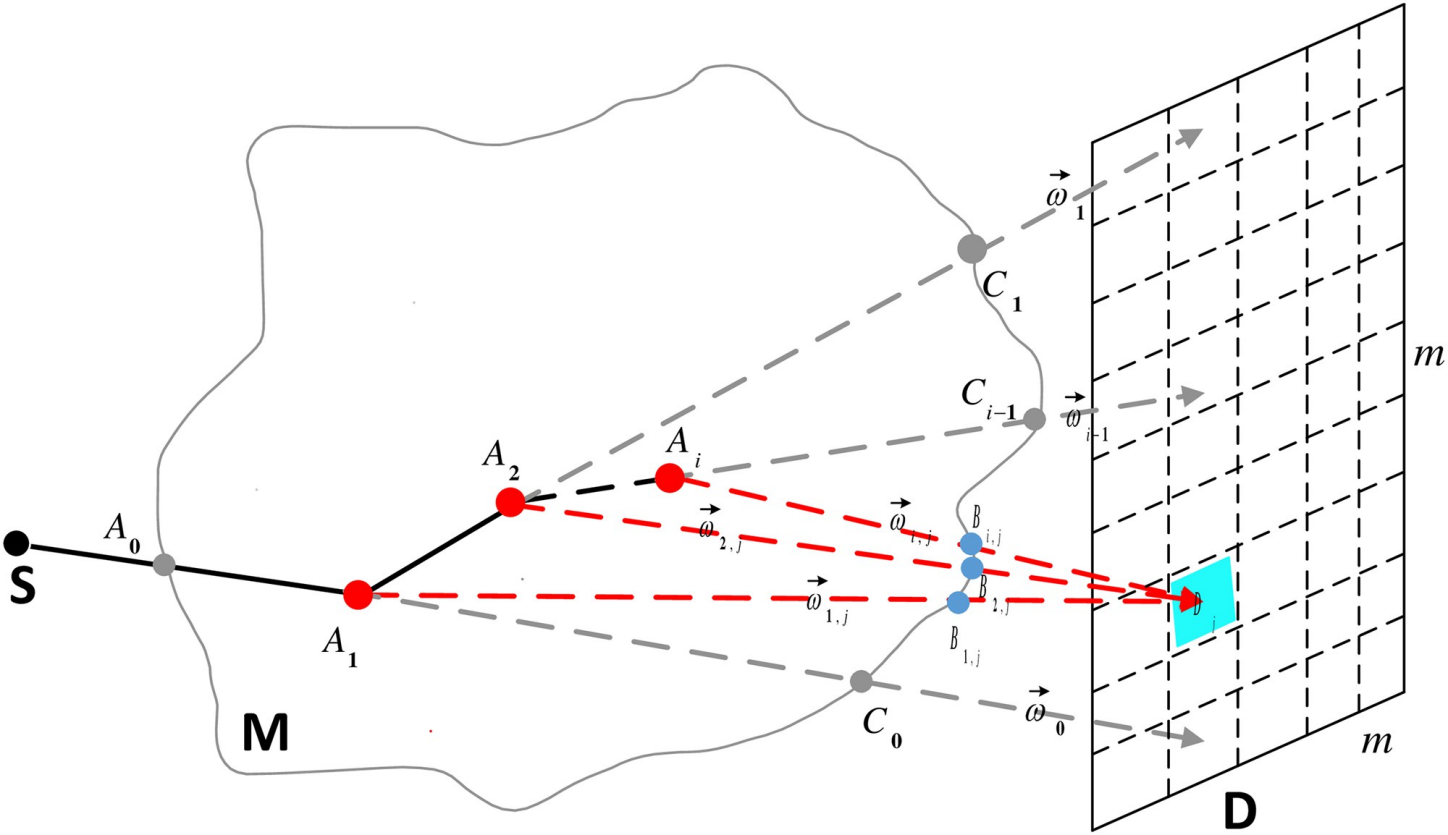
An illustration of the scattered photon paths.

The interaction between photons and the phantom is random, which can be organized overall into three steps: (i) photons are emitted from the X-ray source S and move to the phantom M with a certain probability; (ii) interact with M and scatter, possibly multiple times; (iii) escape M and reach the detector D with a certain probability. Therefore, we introduce the derivation of the path probability *P*(*l*_*i*,*j*_) of scattered photons in three steps. An illustration of the scattered photon paths is shown in Fig 2.

#### Source S to phantom M

Let $I_{0}\left({\vec{\omega }}_{0}\right)$ be the initial flow intensity of a photon emitted from the X-ray source S moving along the unit direction ${\vec{\omega }}_{0}$ and *ϕ*(*E*) is the X-ray energy spectrum distribution. The path length *l* of the photon in M follows the distribution

$$\begin{array}{c} \mu_{tot}\left(x,E\right)\text{exp}[-\int_{l}\mu_{tot}\left(x,E\right)\text{d}x]. \end{array}$$ (2)

So the probability *P*(*S* → *A*_1_) of the photon from the X-ray source S to the first interaction point *A*_1_ along the unit direction ${\vec{\omega }}_{0}$ is

$$\begin{array}{c} \begin{array}{cc} P(\text{S}\to A_{1}) & =\int_{4\pi }\text{d}{\vec{\omega }}_{0}\int_{\Omega_{E_{0}}}\text{d}E_{0}\int_{0}^{c_{0}}\text{d}t_{1}[I_{0}\left({\vec{\omega }}_{0}\right)\phi \left(E_{0}\right)\\ & \times \mu_{tot}\left(A_{1},E_{0}\right)\text{exp}[-\int_{0}^{t_{1}}\mu_{tot}\left(A_{0}+t{\vec{\omega }}_{0},E_{0}\right)\text{d}t]], \end{array} \end{array}$$ (3)

where *E*_0_ is the initial energy of the photon, $\Omega_{E_{0}}$ is the value space of the energy spectrum, $A_{1}=A_{0}+t_{1}{\vec{\omega }}_{0}$, 0 ≤ *t*_1_ ≤ *c*_0_, is the length from *A*_0_ to *A*_1_ along the unit direction ${\vec{\omega }}_{0}$, which is a random variable, *c*_0_ is the length from *A*_0_ to *C*_0_ along the unit direction ${\vec{\omega }}_{0}$ and $C_{0}=A_{0}+c_{0}{\vec{\omega }}_{0}$.

#### Scatter

Each interaction is characterised by the associated linear attenuation coefficient *μ*_*tot*_(***x***, *E*), which represents the probability of photon passing through the unit path and interacting with the phantom M, and is a function of both the spatial coordinates ***x*** and the energy *E*. The quantity *μ*_*tot*_(***x***, *E*) is the sum of $\mu_{T_{0}}\left(x,E\right)$, $\mu_{T_{1}}\left(x,E\right)$ and *μ*_*a*_(***x***, *E*), where $\mu_{T_{0}}\left(x,E\right)$ is the Compton scatter coefficient, $\mu_{T_{1}}\left(x,E\right)$ is the Rayleigh scatter coefficient and *μ*_*a*_(***x***, *E*) is the photoelectric coefficient. The details for X-ray interactions can be found in the reference [22]. The probability *P*(*A*_*i*−1_ → *A*_*i*_|*A*_*i*−2_ → *A*_*i*−1_) that the photon interacts with M at *A*_*i*−1_ (*i* ∈ {2, …, *n*}, *n* is the highest scatter order) and reaches the next-order interaction point *A*_*i*_ is

$$\begin{array}{c} \begin{array}{cc} P(A_{i-1}\to A_{i}|A_{i-2}\to A_{i-1}) & =\sum_{\delta_{i-1}=0}^{1}p_{T_{\delta_{i-1}}}\left(A_{i-1}\right)\int_{4\pi }\text{d}{\vec{\omega }}_{i-1}\int_{0}^{c_{i-1}}\text{d}t_{i}\\ & \times [p_{\theta_{\delta_{i-1}}}\left(A_{i-1},E_{i-2}^{\delta_{i-2}}\to E_{i-1}^{\delta_{i-1}},{\vec{\omega }}_{i-2}\to {\vec{\omega }}_{i-1}\right)\\ & \times \mu_{tot}\left(A_{i},E_{i-1}^{\delta_{i-1}}\right)\text{exp}[-\int_{0}^{t_{i}}\mu_{tot}\left(A_{i-1}+t{\vec{\omega }}_{i-1},E_{i-1}^{\delta_{i-1}}\right)\text{d}t]], \end{array} \end{array}$$ (4)

where $p_{T_{0}}\left(A_{i-1}\right)$ and $p_{T_{1}}\left(A_{i-1}\right)$ are the probabilities of Compton scatter and Rayleigh scatter in the (*i* − 1)-th interaction point *A*_*i*−1_, ${\vec{\omega }}_{i-1}$ is the unit scatter direction of the photon after (*i* − 1)-th scatter at *A*_*i*−1_, *c*_*i*−1_ is the length from *A*_*i*−1_ to *C*_*i*−1_ along the unit direction ${\vec{\omega }}_{i-1}$ and $C_{i-1}=A_{i-1}+c_{i-1}{\vec{\omega }}_{i-1}$. As *i* = 2, *P*(*A*_*i*−1_ → *A*_*i*_|*A*_*i*−2_ → *A*_*i*−1_) is *P*(*A*_1_ → *A*_2_|S → *A*_1_). Moreover,

$$\begin{array}{c} p_{\theta_{0}}\left(A_{i-1},E_{i-2}^{\delta_{i-2}}\to E_{i-1}^{0},{\vec{\omega }}_{i-2}\to {\vec{\omega }}_{i-1}\right)=\frac{\mu_{T_{0}}\left(A_{i-1},E_{i-2}^{\delta_{i-2}}\to E_{i-1}^{0},{\vec{\omega }}_{i-2}\to {\vec{\omega }}_{i-1}\right)}{\mu_{tot}\left(A_{i-1},E_{i-1}^{0}\right)} \end{array}$$ (5)

and

$$\begin{array}{c} p_{\theta_{1}}\left(A_{i-1},E_{i-2}^{\delta_{i-2}}\to E_{i-1}^{1},{\vec{\omega }}_{i-2}\to {\vec{\omega }}_{i-1}\right)=\frac{\mu_{T_{1}}\left(A_{i-1},E_{i-2}^{\delta_{i-2}}\to E_{i-1}^{1},{\vec{\omega }}_{i-2}\to {\vec{\omega }}_{i-1}\right)}{\mu_{tot}\left(A_{i-1},E_{i-1}^{1}\right)} \end{array}$$ (6)

are the probability density function (PDF) of the Compton scatter and Rayleigh scatter polar angle and are normalized linear attenuation coefficients. The quantity $E_{i-1}^{0}$ and $E_{i-1}^{1}$ are the remaining energy at *A*_*i*−1_ after Compton and Rayleigh scatter occurred, respectively.

So the probability *P*(*A*_1_ → ⋯ → *A*_*i*_|S → *A*_1_) that the photon interacts with M at *A*_*i*−1_ (*i* = {2, …, *n*}) and reaches the interaction point *A*_*i*_ is

$$\begin{array}{c} \begin{array}{cc} P(A_{1}\to \cdots \to A_{i}|\text{S}\to A_{1}) & =P\left(A_{1}\to A_{2}|\text{S}\to A_{1}\right)\prod_{k=3}^{i}P\left(A_{k-1}\to A_{k}|A_{k-2}\to A_{k-1}\right)\\ & =\prod_{k=2}^{i}\{\sum_{\delta_{k-1}=0}^{1}p_{T_{\delta_{k-1}}}\left(A_{k-1}\right)\int_{4\pi }\text{d}{\vec{\omega }}_{k-1}\int_{0}^{c_{k-1}}\text{d}t_{k}\\ & \times [p_{\theta_{\delta_{k-1}}}\left(A_{k-1},E_{k-2}^{\delta_{k-2}}\to E_{k-1}^{\delta_{k-1}},{\vec{\omega }}_{k-2}\to {\vec{\omega }}_{k-1}\right)\\ & \times \mu_{tot}\left(A_{k},E_{k-1}^{\delta_{k-1}}\right)\text{exp}[-\int_{0}^{t_{k}}\mu_{tot}\left(A_{k-1}+t{\vec{\omega }}_{k-1},E_{k-1}^{\delta_{k-1}}\right)\text{d}t]]\}. \end{array} \end{array}$$ (7)

#### Interaction point *A*_*i*_ to fixed detector pixel *D*_*j*_

The photon will escape from the phantom M and reach the detector D with a certain probability after *i*-th scatter occurs at *A*_*i*_, *i* = 1, 2, …, *n*. In order to improve the efficiency, only the photons that reach the fixed detector after interaction are considered. This is called fixed forced detection (FFD) [5, 9, 10]. As shown in Fig 2, after the *i*-th interaction occurs at *A*_*i*_, *i* = 1, 2, …, *n*, the photon reaches the fixed detector pixel *D*_*j*_, *j* = 1, …, *m* × *m*, with a certain probability along the forced unit direction ${\vec{\omega }}_{i,j}$. The probability is

$$\begin{array}{c} \begin{array}{cc} P(A_{i}\to D_{j}) & =\sum_{\delta_{i}=0}^{1}p_{T_{\delta_{i}}}\left(A_{i}\right)\int_{\Omega_{A_{i},D_{j}}}\text{d}{\vec{\omega }}_{i,j}[p_{\theta_{\delta_{i}}}\left(A_{i},E_{i-1}^{\delta_{i-1}}\to E_{i}^{\delta_{i}},{\vec{\omega }}_{i-1}\to {\vec{\omega }}_{i,j}\right)\\ & \times \text{exp}[-\int_{0}^{b_{i,j}}\mu_{tot}\left(A_{i}+t{\vec{\omega }}_{i,j},E_{i}^{\delta_{i}}\right)\text{d}t]]\\ & \approx \sum_{\delta_{i}=0}^{1}p_{T_{\delta_{i}}}\left(A_{i}\right)p_{\theta_{\delta_{i}}}\left(A_{i},E_{i-1}^{\delta_{i-1}}\to E_{i}^{\delta_{i}},{\vec{\omega }}_{i-1}\to {\vec{\omega }}_{i,j}\right)\\ & \times \text{exp}[-\int_{0}^{b_{i,j}}\mu_{tot}\left(A_{i}+t{\vec{\omega }}_{i,j},E_{i}^{\delta_{i}}\right)\text{d}t]\Omega_{A_{i},D_{j}}, \end{array} \end{array}$$ (8)

where $\Omega_{A_{i},D_{j}}$ is the solid angle of the detector pixel *D*_*j*_ corresponding to *A*_*i*_, $\Omega_{A_{i},D_{j}}\approx \frac{cos\alpha_{i,j}{h_{D_{j}}}^{2}}{|\vec{A_{i}D_{j}}{|}^{2}}$, $h_{D_{j}}^{2}$ is the area of the detector pixel *D*_*j*_ and *α*_*i*,*j*_ represents the angle between ${\vec{\omega }}_{i,j}$ and the normal direction of the detector pixel *D*_*j*_, $B_{i,j}=A_{i}+b_{i,j}{\vec{\omega }}_{i,j}$ is the intersection of the photon movement along ${\vec{\omega }}_{i,j}$ and M.

So the probability *P*(*l*_*i*,*j*_) that the photon follows the path *l*_*i*,*j*_: S → *A*_0_ → *A*_1_ → ⋯ → *A*_*i*_ → *B*_*i*,*j*_ → *D*_*j*_, *i* ∈ {1, …, *n*}, is

$$\begin{array}{c} \begin{array}{cc} P(l_{i,j}) & =P\left(\text{S}\to A_{1}\to A_{2}\to \cdots \to A_{i}\right)P\left(A_{i}\to D_{j}\right)\\ & =P\left(\text{S}\to A_{1}\right)P\left(A_{1}\to A_{2}|\text{S}\to A_{1}\right)\prod_{k=3}^{i}P\left(A_{k-1}\to A_{k}|A_{k-2}\to A_{k-1}\right)P\left(A_{i}\to D_{j}\right)\\ & =\sum_{\delta_{i}=0}^{1}p_{T_{\delta_{i}}}\left(A_{i}\right)\int_{\Omega_{A_{i},D_{j}}}\text{d}{\vec{\omega }}_{i,j}[p_{\theta_{\delta_{i}}}\left(A_{i},E_{i-1}^{\delta_{i-1}}\to E_{i}^{\delta_{i}},{\vec{\omega }}_{i-1}\to {\vec{\omega }}_{i,j}\right)\\ & \times \text{exp}[-\int_{0}^{b_{i,j}}\mu_{tot}\left(A_{i}+t{\vec{\omega }}_{i,j},E_{i}^{\delta_{i}}\right)\text{d}t]]\\ & \times \prod_{k=2}^{i}\{\sum_{\delta_{k-1}=0}^{1}p_{T_{\delta_{k-1}}}\left(A_{k-1}\right)\int_{4\pi }\text{d}{\vec{\omega }}_{k-1}\int_{0}^{c_{k-1}}\text{d}t_{k}\\ & \times [p_{\theta_{\delta_{k-1}}}\left(A_{k-1},E_{k-2}^{\delta_{k-2}}\to E_{k-1}^{\delta_{k-1}},{\vec{\omega }}_{k-2}\to {\vec{\omega }}_{k-1}\right)\\ & \times \mu_{tot}\left(A_{k},E_{k-1}^{\delta_{k-1}}\right)\text{exp}[-\int_{0}^{t_{k}}\mu_{tot}\left(A_{k-1}+t{\vec{\omega }}_{k-1},E_{k-1}^{\delta_{k-1}}\right)\text{d}t]]\}\\ & \times \int_{4\pi }\text{d}{\vec{\omega }}_{0}\int_{\Omega_{E_{0}}}\text{d}E_{0}\int_{0}^{c_{0}}\text{d}t_{1}[I_{0}\left({\vec{\omega }}_{0}\right)\phi \left(E_{0}\right)\\ & \times \mu_{tot}\left(A_{1},E_{0}\right)\text{exp}[-\int_{0}^{t_{1}}\mu_{tot}\left(A_{0}+t{\vec{\omega }}_{0},E_{0}\right)\text{d}t]]. \end{array} \end{array}$$ (9)

The total probability accumulated after *n*-order interaction in the fixed detector pixel *D*_*j*_, *j* = 1, …, *m* × *m*, is $\sum_{i=1}^{n}P\left(l_{i,j}\right)$.

### gQMCFFD algorithm and implementation

In this section, we give the specific implementation steps for simulating the path probability for scattered photons. QMC and FFD are used in the simulation, we call these simulation steps the gQMCFFD algorithm. We will use Sobol’ points [23, 24] in our simulation. Sobol’ sequences are low-discrepancy sequences with better uniformity than random sequences. Other low-discrepancy sequences, such as Halton sequences [25] and Faure sequences [26], can also be used for simulation.

**Fig 3:**
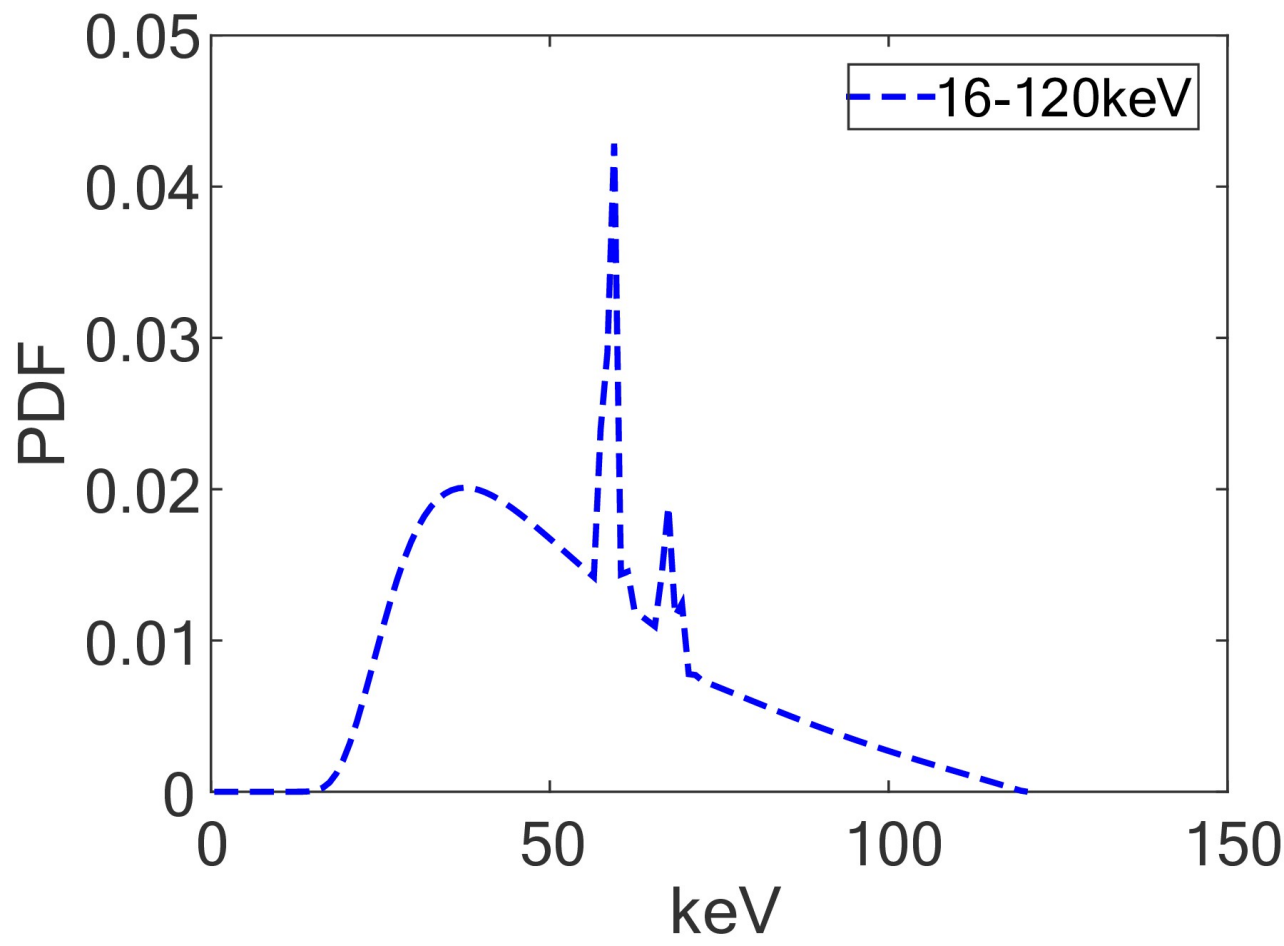
An illustration of photon energy distribution.

The first component *u*_1_ of the pregenerated 4*n*-dimensional Sobol’ point ***u*** = (*u*_1_, *u*_2_, …, *u*_4*n*_) is used to sample the initial energy *E*_0_ from the predetermined energy distribution *ϕ*(*E*_0_) by the Walker’s aliasing method [27]. Walker’s aliasing method is suitable for sampling discrete data and requires only one variable to be sampled. The distribution is shown in Fig 3. The quantity $I_{0}\left({\vec{\omega }}_{0}\right)$ is the initial photon flux along the initial unit direction ${\vec{\omega }}_{0}$ and may be complicated in the solid angle of the photon flux, we use *u*_2_, *u*_3_ to sample uniformly in [0, 4*π*]. Then the unit incident direction ${\vec{\omega }}_{0}$, *I*_0_(*u*_2_, *u*_3_) and the intersection *A*_0_ of the initial incident X-ray and the phantom M can be calculated. So the photon with weight *W*_0_ = *I*_0_(*u*_2_, *u*_3_) starts at *A*_0_ and moves along ${\vec{\omega }}_{0}$ in the phantom M.

The path length *t*_*i*_, *i* = 1, …, *n*, of a photon from its current position *A*_*i*−1_ to the site of the next interaction *A*_*i*_ follows the distribution (see [3])

$$\begin{array}{c} \mu_{tot}\left(A_{i},E_{i-1}^{\delta_{i-1}}\right)\text{exp}[-\int_{0}^{t_{i}}\mu_{tot}\left(A_{i-1}+t{\vec{\omega }}_{i-1},E_{i-1}^{\delta_{i-1}}\right)\text{d}t], \end{array}$$ (10)

where $A_{i}=A_{i-1}+t_{i}{\vec{\omega }}_{i-1}$, ${\vec{\omega }}_{i-1}$ is the unit scatter direction at *A*_*i*−1_, *δ*_*i*−1_ = 0, 1.

So the probability of the photon starting from *A*_*i*−1_, *i* = 1, …, *n*, escaping the phantom M along the unit scatter direction ${\vec{\omega }}_{i-1}$ is

$$\begin{array}{c} p_{i-1}\left({\vec{\omega }}_{i-1},E_{i-1}^{\delta_{i-1}}\right)=\text{exp}[-\int_{0}^{c_{i-1}}\mu_{tot}\left(A_{i-1}+t{\vec{\omega }}_{i-1},E_{i-1}^{\delta_{i-1}}\right)\text{d}t], \end{array}$$ (11)

where $C_{i-1}=A_{i-1}+c_{i-1}{\vec{\omega }}_{i-1}$, *C*_*i*−1_ is the intersection point of the photon along ${\vec{\omega }}_{i-1}$ on the boundary of the phantom M, and *c*_*i*−1_ is the lengh of *A*_*i*−1_ to *C*_*i*−1_, as shown in Fig 2. The primary intensity (namely the probability of the photon starting from S escaping the phantom M along the unit incident direction ${\vec{\omega }}_{0}$ and reaching the detector D) is

$$\begin{array}{c} P_{0}\left(\text{S}\to A_{0}\to C_{0}\to \text{D}\right)=W_{0}p_{0}\left({\vec{\omega }}_{0},E_{0}\right). \end{array}$$ (12)

After the (*i* − 1)-th interaction point *A*_*i*−1_(*i* = 2, …, *n*) is sampled, we use the component *u*_4(*i*−1)+1_ to sample scatter type by importance sampling [21] and use *u*_4(*i*−1)+2_ and *u*_4(*i*−1)+3_ to sample the unit scatter direction ${\vec{\omega }}_{i-1}$ by the Rational Inverse Transform with Aliasing (RITA) algorithm [21, 28]. Importance sampling attempts to give more weight to important outcomes thereby increasing sampling efficiency and RITA is a general numerical algorithm for random sampling from continuous distributions using the inverse-transform method. Therefore, the photon with weight $W_{i}=W_{i-1}(1-p_{i-1}\left({\vec{\omega }}_{i-1},E_{i-1}^{\delta_{i-1}}\right))$ undergoes the *i*-th interaction inside M, and the *i*-th interaction point *A*_*i*_, *i* = 1, 2, …, *n*, can be calculated by $A_{i}=A_{i-1}+t_{i}{\vec{\omega }}_{i-1}$, the random variable *t*_*i*_ is found by

$$\begin{array}{c} 1-\text{exp}[-\int_{0}^{t_{i}}\mu_{tot}\left(A_{i-1}+t{\vec{\omega }}_{i-1},E_{i-1}^{\delta_{i-1}}\right)\text{d}t]=(1-p_{i-1}\left({\vec{\omega }}_{i-1},E_{i-1}^{\delta_{i-1}}\right))u_{4(i-1)+4}, \end{array}$$ (13)

where *u*_4(*i*−1)+4_ is the (4(*i* − 1) + 4)-th component of 4*n*-dimensional Sobol’ point ***u***.

So the probability that the photon follows the path *l*_*i*,*j*_: S → *A*_0_ → *A*_1_ → ⋯ → *A*_*i*_ → *B*_*i*,*j*_ → *D*_*j*_, *i* ∈ {1, …, *n*}, is

$$\begin{array}{c} P\left(l_{i,j}\right)=W_{i}P_{i}\left(A_{i}\to D_{j}\right). \end{array}$$ (14)

The components *u*_1_, *u*_2_, *u*_3_, *u*_4_ of ***u*** are used to calculate the probability of the path: S → *A*_0_ → *A*_1_. So *P*(*l*_1,*j*_) can be written as

$$\begin{array}{c} P\left(l_{1,j}\right)\;≔\;P_{1}\left(A_{1}\to D_{j}\right)g_{0}\left(u_{1},\ldots ,u_{4}\right). \end{array}$$ (15)

Besides, the photon path S →*A*_0_ → *A*_1_ → ⋯ → *A*_*i*_ is a Markov chain, the photon path S → *A*_0_ → *A*_1_ is sampled by *u*_1_, *u*_2_, *u*_3_, *u*_4_, *A*_*i*−1_ → *A*_*i*_, *i* = 2, …, *n*, is sampled by *u*_4(*i*−1)+1_, *u*_4(*i*−1)+2_, *u*_4(*i*−1)+3_ and *u*_4(*i*−1)+4_ and *A*_*i*_ is only related with *A*_*i*−1_. So

$$\begin{array}{c} \begin{array}{cc} P(l_{i,j}) & =W_{i}P_{i}\left(A_{i}\to D_{j}\right)\\ & ≔P_{i}\left(A_{i}\to D_{j}\right)g_{0}\left(u_{1},\ldots ,u_{4}\right)\prod_{k=1}^{i-1}g_{k}\left(u_{4(k-1)+1},\ldots ,u_{4k+4}\right). \end{array} \end{array}$$ (16)

The total probability accumulated after *n*-order interaction in the fixed detector pixel *D*_*j*_, *j* = 1, …, *m* × *m*, is

$$\begin{array}{c} \begin{array}{cc} f_{n,j}\left(u\right) & ≔\sum_{i=1}^{n}P\left(l_{i,j}\right)\\ & =\sum_{i=1}^{n}W_{i}P_{i}\left(A_{i}\to D_{j}\right)\\ & =g_{0}\left(u_{1},\ldots ,u_{4}\right)\\ & \times (P_{1}\left(A_{1}\to D_{j}\right)+\sum_{i=2}^{n}\prod_{k=1}^{i-1}P_{i}\left(A_{i}\to D_{j}\right)g_{k}\left(u_{4(k-1)+1},\ldots ,u_{4k+4}\right)). \end{array} \end{array}$$ (17)

So *f*_*n*,*j*_ is a function depending on ***u***, where ***u*** = (*u*_1_, *u*_2_, …, *u*_4*n*_), and the formal dimension of the scatter correction mechanism is *d* = 4*n*. If low discrepancy points [19, 20] are used for sampling, the mechanism is called as gQMCFFD algorithm. In this paper, Sobol’ sequences [23, 24] are used for sampling. If random sequences are used, the corresponding mechanism is called as gMCFFD algorithm.

In summary, the new scatter correction mechanism can be summarized in Algorithm 1.

**Algorithm 1** gQMCFFD algorithm flow in one simulation

1: Input: For a given scatter order *n*, input a 4*n*-dimensional Sobol’ point (*u*_1_, …, *u*_4*n*_).

2: *u*_1_ is used to sample *E*_0_ from *ϕ*(*E*_0_) by the inverse transform method; *u*_2_ and *u*_3_ are used to sample the unit incident direction ${\vec{\omega }}_{0}$ and acquire the flow intensity *W*_0_ = *I*_0_(*u*_2_, *u*_3_) at *A*_0_ along ${\vec{\omega }}_{0}$. Set *i* = 1. *u*_4_ is used to sample the first scatter point *A*_1_ by Eq (13).

3: If *i* ≥ *n*, go to step 5; otherwise, set *i* = *i* + 1, using *u*_4(*i*−1)+1_ to sample scatter type and using *u*_4(*i*−1)+2_, *u*_4(*i*−1)+3_ to sample the unit scatter direction ${\vec{\omega }}_{i-1}$ at *A*_*i*−1_, *i* = 2, …, *n* by RITA. Calculate $p_{i-1}\left({\vec{\omega }}_{i-1},E_{i-1}^{\delta_{i-1}}\right)$ and $E_{i-1}^{\delta_{i-1}},\delta_{i}=0,1$.

4: *u*_4(*i*−1)+4_ are used to sample scatter point *A*_*i*_ by Eq (13).

5: Let $W_{i}=W_{i-1}(1-p_{i-1}\left({\vec{\omega }}_{i-1},E_{i-1}^{\delta_{i-1}}\right))$, calculate *P*(*l*_*i*,*j*_) = *W*_*i*_*P*_*i*_(*A*_*i*_ → *D*_*j*_). If *i* < *n*, go to step 3; otherwise, go to step 6.

6: Returns *P*(*l*_*i*,*j*_), *i* = 1, …, *n*, *j* = 1, …, *m* × *m*, where *m* × *m* is the number of detector pixels.

The QMC or MC estimate of the total probability $\sum_{i=1}^{n}P\left(l_{i,j}\right)$ is of the form

$$\begin{array}{c} {\hat{I}}_{N,d}\left(f_{n,j}\right)=\frac{1}{N}\sum_{i=1}^{N}f_{n,j}\left(u^{i}\right), \end{array}$$ (18)

where $u^{i}=\left\{u_{1}^{i},\ldots ,u_{4n}^{i}\right\},i=1,2,\ldots ,N$, are Sobol’ points over [0, 1]^*d*^ for QMC or random samples of uniform distribution for MC. For fixed *n* and *j*, the QMC or MC estimate of $\sum_{i=1}^{n}P\left(l_{i,j}\right)$ is the mathematical expectation $E(f_{n,j}\left(u\right))$.

## Numerical experiments

In this section, we first calculate the photon scatter intensities reaching the detector pixel *D*_256×256_ on homogeneous Al phantom, Bone-Tissue (BT) cylinder, Shepp-Logan phantom (SL) [29] and Head phantom to demonstrate the proportion of higher order scatter will decrease. The detector D is composed of 512 × 512 detector pixels, and the detector pixels are denoted as *D*_*j*_, *j* = 1, …, 512 × 512. *D*_256×256_ is the center of the detector D. Secondly, we calculate the X-ray scatter intensities reaching the whole detector D and compare gQMCFFD, gMCFFD, gQMCFRD [21] with gMCFRD [21], campare gQMCFFD with SKS [6] and fASKS [7]. In order to improve efficiency, sparse matrix method is used. The 64 × 64 matrices with interpolation are used instead of 512 × 512 matrices in a simulation. The nomenclatures of different algorithm acronyms are shown in Table 1.

**Table 1:** The different algorithm acronyms.

Abbreviations	Nomenclature
gMCFRD	graphics processing unit-based Monte Carlo with forced random detection
gQMCFRD	graphics processing unit-based quasi-Monte Carlo with forced random detection
gMCFFD	graphics processing unit-based Monte Carlo with forced fixed detection
gQMCFFD	graphics processing unit-based quasi-Monte Carlo with forced fixed detection
MC-GPU	Monte Carlo-graphics processing unit
SKS	scatter kernel superposition
fASKS	fast adaptive scatter kernel superposition

**Fig 4:**
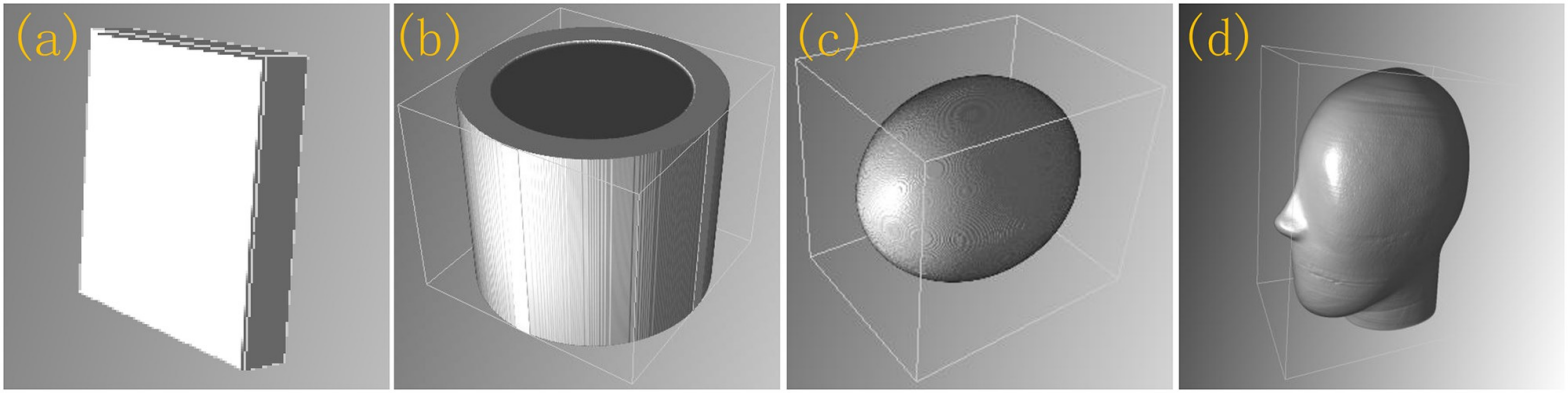
(a) homogeneous Al phantom; (b) Bone-Tissue (BT) cylinder; (c) Shepp-Logan (SL) phantom; (d) Head phantom.

The homogeneous Al phantom (which is composed of aluminium) has dimension 160 × 28 × 160 voxels with a voxel size of 1 × 1 × 1mm^3^. BT cylinder (which is composed of bone, tissue and air) has dimension *π* × 60^2^ × 108 voxels with a voxel size of 0.5 × 0.5 × 0.5mm^3^. The thickness of the bone tube is 5mm, the volume is *π* × (30^2^ − 25^2^) × 54mm^3^. The volume of the tissue is *π* × 25^2^ × 54mm^3^. SL phantom (which is composed of bone, water and air) has dimension 320 × 400 × 360 voxels with a voxel size of 0.5 × 0.5 × 0.5mm^3^. Head phantom (which is composed of tissue, bone and air) has dimension 270 × 400 × 430 voxels with a voxel size of 0.5 × 0.5 × 0.5mm^3^. Fig 4(a)–4(d) is the geometric illustration of homogeneous Al phantom, BT cylinder, SL phantom, Head phantom, respectively.

In our numerical experiments, the X-ray source S is a point source with energy spectrum 16 − 120keV. The energy spectrum distribution is shown in Fig 3. The X-ray source S-to-phantom M distance and phantom M-to-detector D distance are both 500mm. The detector D resolution is 512 × 512 pixels with a pixel size of 0.8 × 0.8mm^2^. We use a GeForce RTX 2080 Ti graphics card that is equipped with 4352 processors and 11.0 GB global memory as computational hardware. In simulations, PENELOPE physical database of Geant4 (GEometry ANd Tracking) [30] is employed.

To measure accuracy, we compute the relative difference (RD), e.g., ||*r* − *t*||_2_/||*r*||_2_, where *t* is the scatter intensity computed by gQMCFRD, gMCFFD or gQMCFFD, and *r* is that computed by gMCFRD [21] or MC-GPU [11], ${\left||\gamma |\right|}_{2}=\sqrt{\sum_{i=1}^{m\times m}{\gamma_{i}}^{2}},m=512$. To compare the numerical efficiency between gQMCFFD (or gMCFFD, or gQMCFRD) and gMCFRD, we use the so-called figure of merit (FOM) [31]

$$\begin{array}{c} \text{FOM}=\frac{1}{T\sigma^{2}}, \end{array}$$ (19)

where *σ* is the standard deviation and *T* (in minutes) is defined as the calculation time and the efficiency improvement factor (EIF)

$$\begin{array}{c} \text{EIF}=\frac{{FOM}_{\text{A}}}{{FOM}_{\text{gMCFRD}}}, \end{array}$$ (20)

where A is the gQMCFFD (or gMCFFD, or gQMCFRD) algorithm. An EIF greater than 1 implies algorithm A is more efficient relative to gMCFRD. In a simulation, gMCFRD and gQMCFRD use 2^29^ source photons, gMCFFD and gQMCFFD use 2^15^ source photons.

### The proportion of *i* order scatter intensities

Table 2 shows the probability *P*(*l*_*i*,256×256_) of each *i*-order scattered path, namely, the *i*-order scatter intensity that a photon reaches a fixed detector pixel *D*_256×256_ after exactly *i*-order scatter occurs. In addition, it shows the proportion of the probability *P*(*l*_*i*,256×256_) of each *i*-order scattered path to the total probability *f*_*n*,256×256_, where $f_{n,256\times 256}=\sum_{i=1}^{n}P\left(l_{i,256\times 256}\right)$ is the total probability accumulated after *n*-order interaction in the fixed detector pixel *D*_256×256_. It can be seen that *P*(*l*_*i*,256×256_) is almost 0 for homogeneous Al phantom and Bone-Tissue cylinder as order *i* > 10, and for SL phantom and Head phantom as order *i* > 12 and the *f*_*n*,256×256_ converges to 0.00892 for the homogeneous Al phantom, to 0.00960 for BT cylinder, to 0.00876 for SL phantom, to 0.01209 for the Head phantom. So we take the maximum scatter order *n* = 10 for homogeneous Al phantom and Bone-Tissue cylinder and *n* = 15 for SL phantom and Head phantom in simulations.

**Table 2:** The probability *P*(*l*_*i*,256×256_) of *i*-order scattered path.

i	Al	BT	SL	Head
1	5.00 × 10^−3^	5.15 × 10^−3^	3.66 × 10^−3^	5.51 × 10^−3^
2	2.26 × 10^−3^	2.89 × 10^−3^	2.24 × 10^−3^	2.91 × 10^−3^
3	9.63 × 10^−4^	1.01 × 10^−3^	1.29 × 10^−3^	1.63 × 10^−3^
4	4.15 × 10^−4^	3.60 × 10^−4^	7.46 × 10^−4^	9.48 × 10^−5^
5	1.72 × 10^−4^	1.25 × 10^−4^	4.19 × 10^−4^	5.35 × 10^−4^
6	6.85 × 10^−5^	4.13 × 10^−5^	2.23 × 10^−4^	2.89 × 10^−4^
7	2.69 × 10^−5^	1.31 × 10^−5^	1.11 × 10^−4^	1.47 × 10^−4^
8	9.76 × 10^−6^	3.84 × 10^−6^	5.29 × 10^−5^	7.09 × 10^−5^
9	3.20 × 10^−6^	1.09 × 10^−6^	2.19 × 10^−5^	3.18 × 10^−5^
10	1.12 × 10^−6^	2.92 × 10^−7^	9.81 × 10^−6^	1.37 × 10^−5^
11	3.80 × 10^−7^	7.70 × 10^−8^	3.63 × 10^−6^	5.53 × 10^−6^
12	1.18 × 10^−7^	1.90 × 10^−8^	1.08 × 10^−6^	2.52 × 10^−6^
Sum	8.92 × 10^−3^	9.60 × 10^−3^	8.76 × 10^−3^	1.209 × 10^−2^

Notes: *N* = 2^17^.

### Scatter intensities

**Fig 5:**
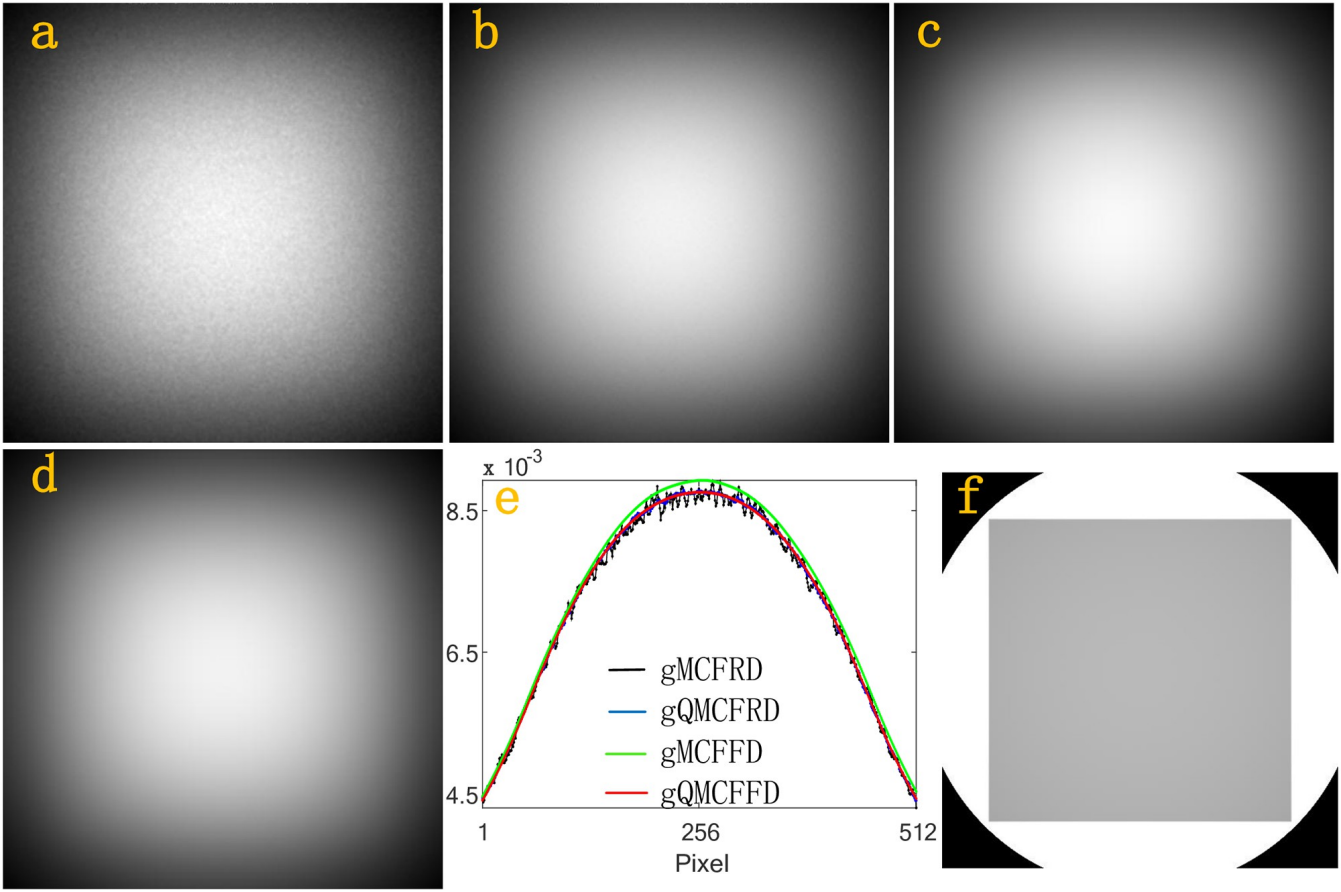
(a)-(d) total scatter intensities of the Al phantom by gMCFRD, gQMCFRD, gMCFFD and gQMCFFD; (e) is the scatter intensity profiles of Al phantom through the center of the corresponding detector which parallel to the x-axis; (f) The primary intensities.

**Fig 6:**
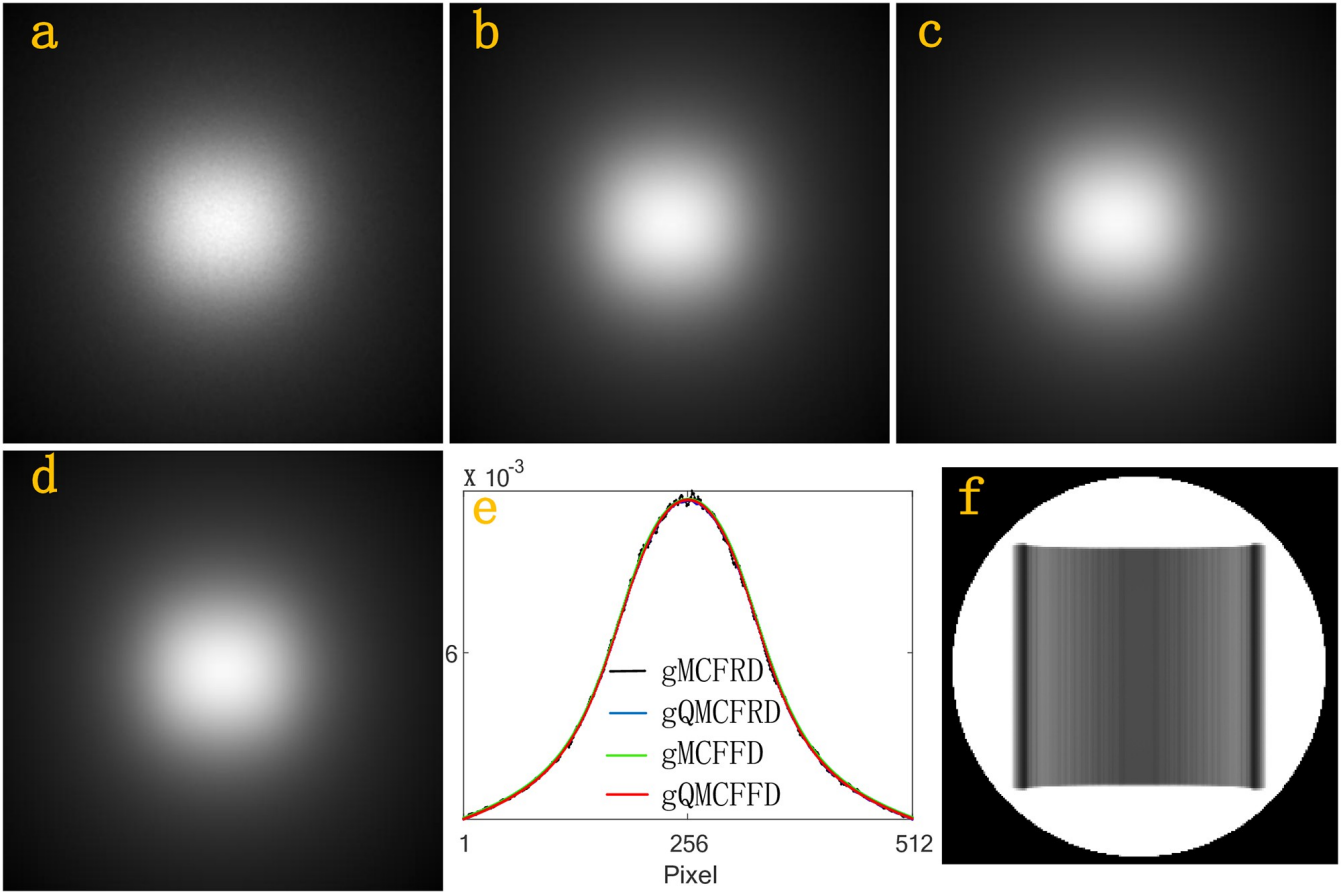
(a)-(d) total scatter intensities of the BT by gMCFRD, gQMCFRD, gMCFFD and gQMCFFD; (e) is the scatter intensity profiles of BT phantom through the center of the corresponding detector which parallel to the x-axis; (f) The primary intensities.

**Fig 7:**
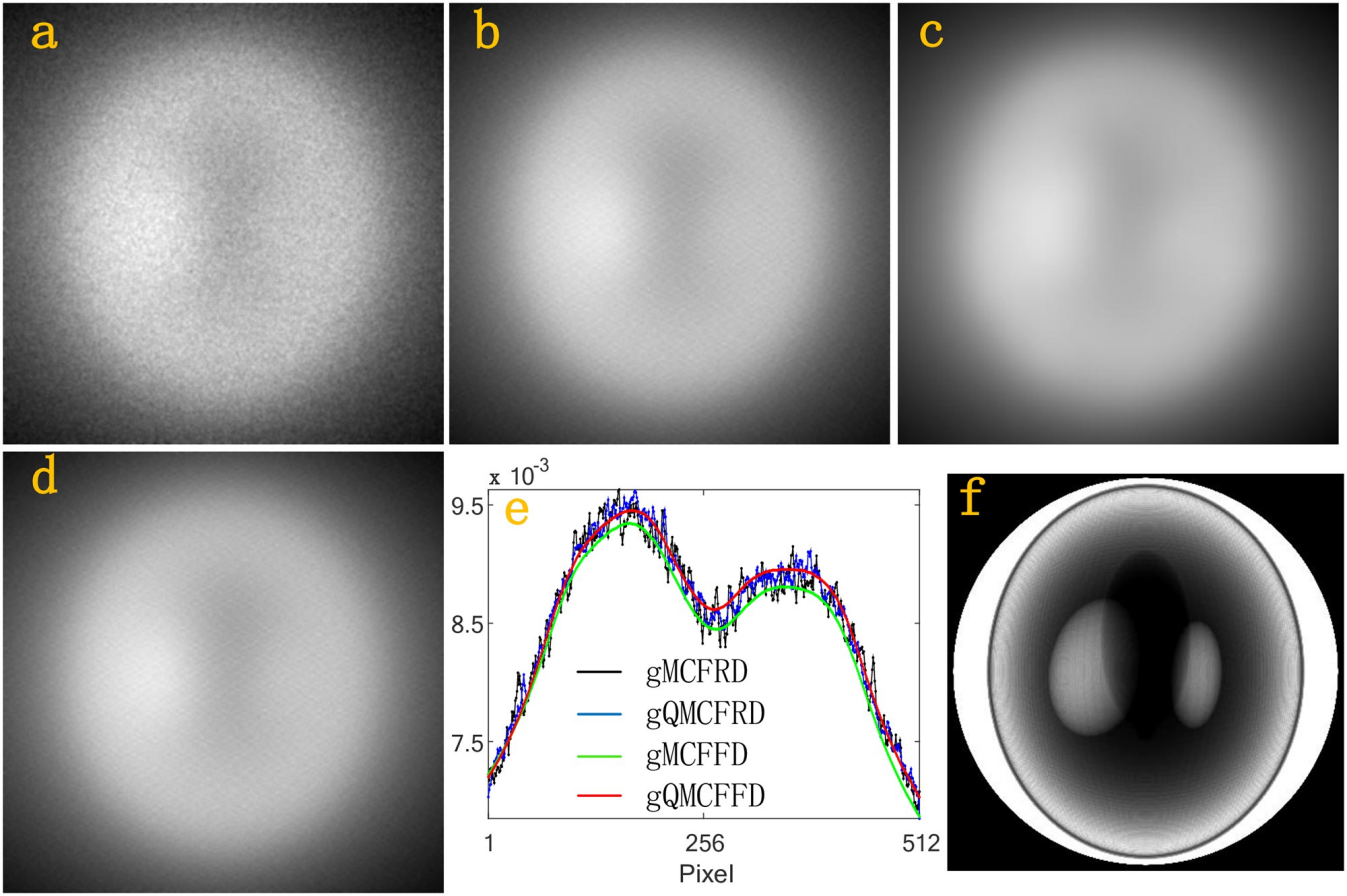
(a)-(d) total scatter intensities of the SL by gMCFRD, gQMCFRD, gMCFFD and gQMCFFD; (e) is the scatter intensity profiles of SL phantom through the center of the corresponding detector which parallel to the x-axis; (f) The primary intensities.

**Fig 8:**
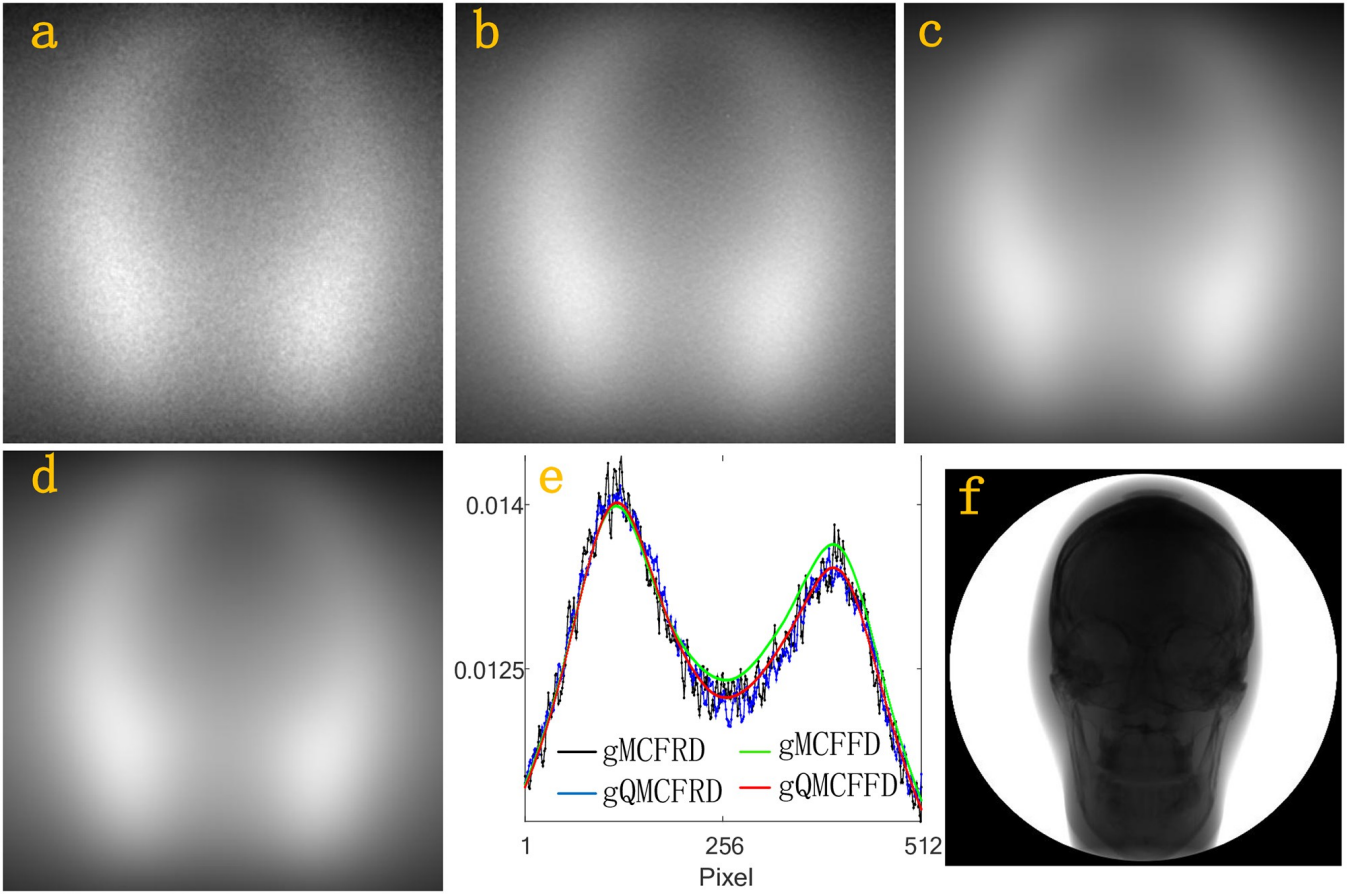
(a)-(d) total scatter intensities of the Head by gMCFRD, gQMCFRD, gMCFFD and gQMCFFD; (e) is the scatter intensity profiles of Head phantom through the center of the corresponding detector which parallel to the x-axis; (f) The primary intensities.

Next, we simulate the scatter intensities *f*_*n*,*j*_, *j* = 1, …, 512 × 512 at the whole detector D. In a simulation, gMCFRD, gQMCFRD, gMCFFD, gQMCFFD uses 2^29^, 2^29^, 2^15^ and 2^15^ source photons, respectively. Fig 5(a)–5(d), which is generated by a 512 × 512 matrix, is the scatter intensities received by the whole detector D after the photon interacts with the homogeneous Al phantom using gMCFRD, gQMCFRD, gMCFFD, gQMCFFD, respectively. Fig 5(e) is the scatter intensity profiles of Al phantom through the center of the corresponding detector which parallel to the x-axis, where the black profile, the blue profile, the green profile, the red profile, is calculated by gMCFRD, gQMCFRD, gMCFFD, gQMCFFD, respectively. Fig 5(f) is the primary intensities. Figs 6–8, are the results of BT cylinder, SL phantom, Head, respectively. The resulting images which simulated by gMCFRD, gQMCFRD, gMCFFD and gQMCFFD are in good agreement, indicating the accuracy of gQMCFFD. As the resulting images are in good agreement, the number of photons used by gQMCFFD in a simulation is only $\frac{1}{16384}$ times the number of photons used by gQMCFRD, which also further reflects the advantages of gQMCFFD.

Table 3 shows the running time T (min), standard deviation *σ* (running one hundred times) of gMCFRD running 2^29^ photons, gQMCFRD running 2^29^ photons, gMCFFD running 2^15^ photons, gQMCFFD running 2^15^ photons for homogeneous Al phantom and the relative difference (RD) and EIF which compared gQMCFRD, gMCFFD, gQMCFFD with gMCFRD. Tables 4–6 show the results for Bone-Tissue cylinder, Shepp-Logan phantom and Head, respectively. It can be seen from Table 3 that the RD of gQMCFFD is 1.17% and the EIF of gQMCFFD is 46.04 with compared to gMCFRD for Al phantom. For BT cylinder, SL phantom and Head phantom, the RD of gQMCFFD is 1.32%, 1.37% and 1.33%, the EIF of gQMCFFD is 38.51, 3.96 and 4.01, respectively. In addition, the EIF of gQMCFFD is much greater than 1 with compared to gQMCFRD and gMCFFD.

**Table 3:** The total scatter intensity results of gQMCFRD, gMCFFD and gQMCFFD are compared with the results of gMCFRD for Al phantom.

	*log*_2_(*N*)	T (min)	RD	*σ*	FOM	EIF
gMCFRD	29	1.51	\	1.14 × 10^−2^	5096	\
gQMCFRD	29	1.83	1.22%	2.38 × 10^−3^	96470	18.93
gMCFFD	15	0.44	1.29%	1.14 × 10^−2^	17488	3.43
gQMCFFD	15	0.48	1.17%	2.98 × 10^−3^	234599	46.04

**Table 4:** The total scatter intensity results of gQMCFRD, gMCFFD and gQMCFFD are compared with the results of gMCFRD for BT phantom.

	*log*_2_(*N*)	T (min)	RD	*σ*	FOM	EIF
gMCFRD	29	2.73	\	7.94 × 10^−3^	5810	\
gQMCFRD	29	3.25	0.86%	2.10 × 10^−3^	69772	12.01
gMCFFD	15	1.29	0.95%	7.36 × 10^−3^	14311	2.46
gQMCFFD	15	1.32	0.85%	1.84 × 10^−3^	223764	38.51

**Table 5:** The total scatter intensity results of gQMCFRD, gMCFFD and gQMCFFD are compared with the results of gMCFRD for SL phantom.

	*log*_2_(*N*)	T (min)	RD	*σ*	FOM	EIF
gMCFRD	29	19.31	\	1.28 × 10^−2^	316	\
gQMCFRD	29	24.75	1.50%	7.37 × 10^−3^	744	2.35
gMCFFD	15	11.82	1.51%	1.44 × 10^−2^	408	1.29
gQMCFFD	15	11.83	1.37%	8.22 × 10^−3^	1251	3.96

**Table 6:** The total scatter intensity results of gQMCFRD, gMCFFD and gQMCFFD are compared with the results of gMCFRD for Head phantom.

	*log*_2_(*N*)	T (min)	RD	*σ*	FOM	EIF
gMCFRD	29	15.17	\	1.17 × 10^−2^	482	\
gQMCFRD	29	18.73	1.38%	6.26 × 10^−3^	1362	2.83
gMCFFD	15	8.55	1.68%	1.35 × 10^−2^	642	1.33
gQMCFFD	15	8.63	1.33%	7.74 × 10^−3^	1934	4.01

### Validation with MC-GPU and SKS

**Fig 9:**
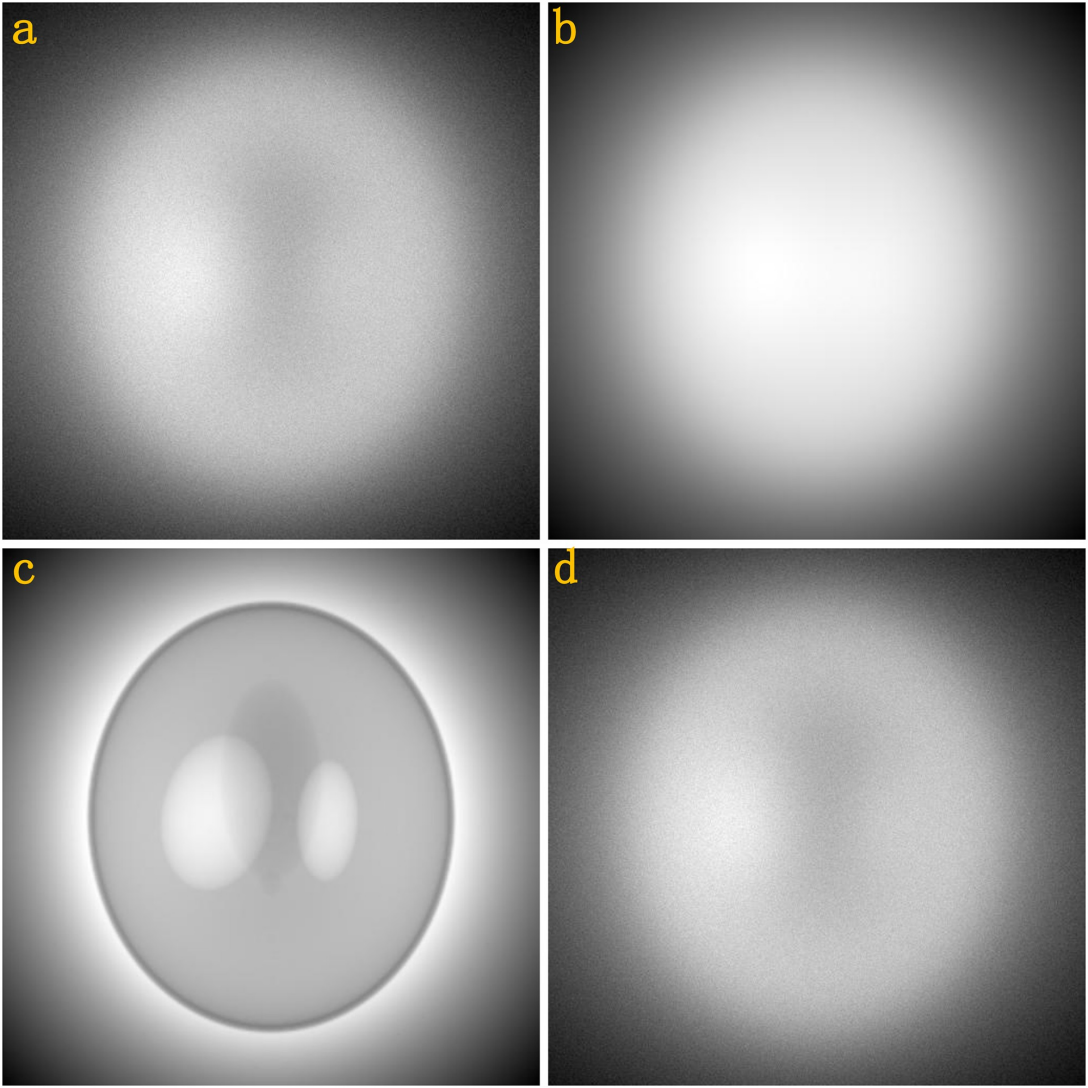
(a)-(d) total scatter intensities of the SL by MC-GPU, SKS, fASKS and gQMCFRD.

**Fig 10:**
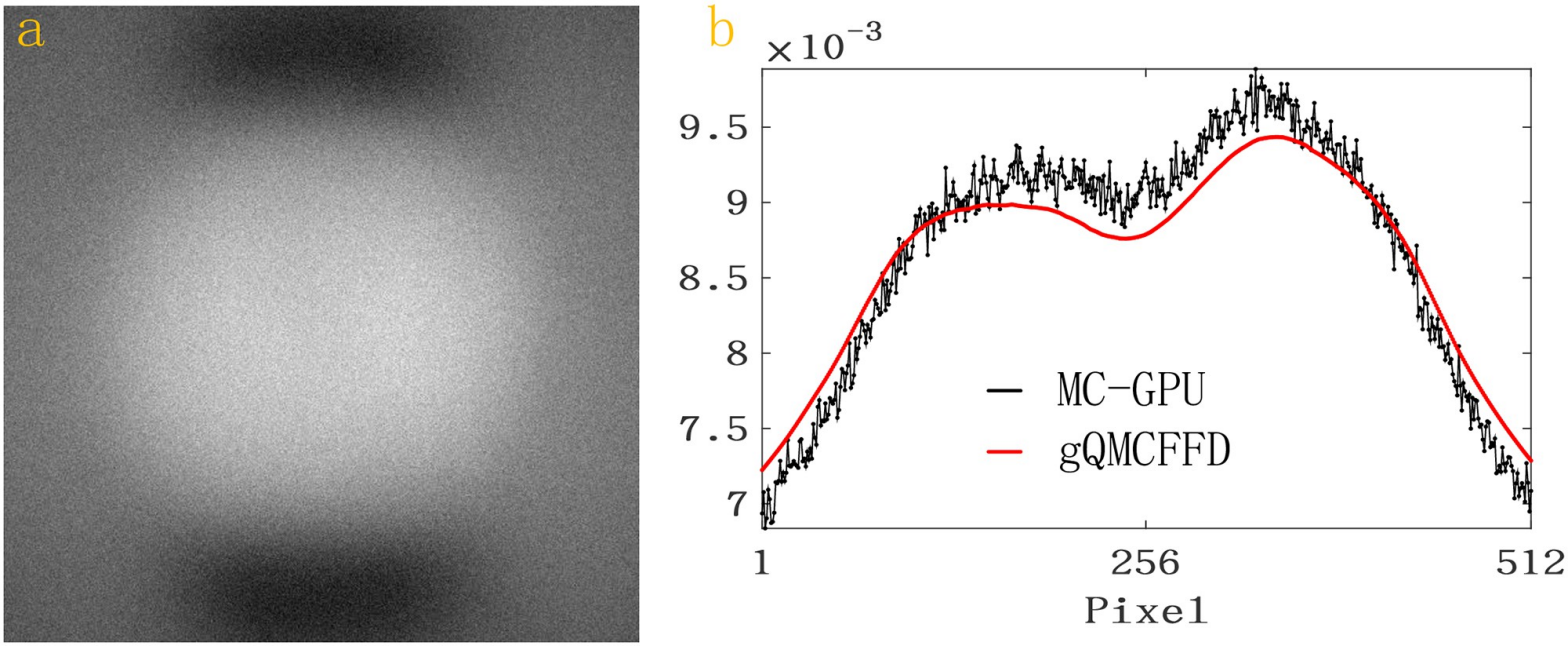
(a) is the difference image between the scatter estimates of SL for gQMCFFD and MC-GPU; (b) is the scatter intensity profiles of SL through the center of the corresponding detector which parallel to the x-axis.

Fig 9(a)–9(d) is the scatter intensities after the photon interacts with the SL using MC-GPU [11], SKS [6], fASKS [7], gQMCFFD respectively. Visually, Fig 9(a) and 9(d) are in good agreement. When combining gQMCFFD with the sparse matrix method and running on the GeForce RTX 2080 Ti graphics card, the simulation time of scatter intensity at one angle of the SL phantom can be reduced to 2 seconds and the RD is 3.53% with compared to MC-GPU. The RDs of SKS and fASKS are also present in Table 7. Consistent with the results in Fig 9, SKS and fASKS have relatively large RDs. The RD of SKS is 11.23% and The RD of fASKS is 7.85%. Fig 10(a) is the difference image between the scatter estimates of SL for gQMCFFD and MC-GPU; (b) is the scatter intensity profiles of SL at midline, where the black profile is estimated by MC-GPU, the red profile is estimated by gQMCFFD.

**Table 7:** The total scatter intensity results of SKS, fASKS and gQMCFFD are compared with the results of MC-GPU for SL phantom.

	MC-GPU	SKS	fASKS	gQMCFFD
*log*_2_(*N*)	38	\	\	15
RD	\	11.23%	7.85%	3.53%

## Conclusions

The goals of the paper are to develop and study a new and efficient scatter estimation and correction mechanism for CT. Firstly, the path probability of the interaction between the photon and the phantom is transformed into a high-dimensional integral. Secondly, an efficient scatter estimation algorithm is proposed. We verified the effectiveness and robustness of the gQMCFFD algorithm in homogeneous Al phantom, BT cylinder, SL phantom and Head phantom, and found that gQMCFFD is more successful than gMCFRD. The results are in excellent agreement with RDs less than 1.5% and EIFs are 4 ∼ 46. Finally, we compare gQMCFFD, SKS, fASKS with MC-GPU in the SL phantom to further illustrate the effectiveness and robustness of gQMCFFD. By combining gQMCFFD with the sparse matrix method, the simulation time reduces to 2 seconds and the RD is 3.53%.

In this paper, we have transformed the path probability of photons interacting with the phantom into a high-dimensional integral and the high dimensional integral can be solved using QMC simulation and FFD implemented on GPU, with validation against MC simulations. In the future, we will discuss modeling a clinical cone-beam CT (CBCT) system with gQMCFFD and sparse simulation and using the output to remove the scatter intensity from the projection data before reconstruction.
